# Unravelling EFL teachers' mastery of TPACK: Technological pedagogical and content knowledge in writing classes

**DOI:** 10.1016/j.heliyon.2023.e17348

**Published:** 2023-06-15

**Authors:** Hanadi Abubakir, Yousef Alshaboul

**Affiliations:** a National Center for Educational Development, College of Education, Qatar University, Doha, Qatar; b Department of Educational Sciences, College of Education, Qatar University, Doha, Qatar

**Keywords:** Technological pedagogical and content knowledge, TPACK, Teaching writing, EFL teachers, Qatar

## Abstract

Integrating technology into English writing instruction has become integral to improving students' writing skills. However, there is a dearth of research addressing teachers' Technological Pedagogical and Content Knowledge (TPACK) in writing classes. Hence, this study explored preparatory English as a Foreign Language (EFL) teachers' mastery of TPACK in writing classes in Qatar. Using a descriptive approach, one hundred eighty-two in-service teachers responded to a self-reported TPACK survey. According to the findings, teachers' knowledge across all TPACK constructs was high. While teachers' content knowledge was the highest among the four domains, teachers' TPACK was the lowest. Significantly, male teachers showed a greater level of technological knowledge than female teachers. In addition, results show that teachers with 1 to 5 years of experience scored at the highest level of technological knowledge, and teachers who received professional development outperformed their peers in TPACK. The study findings provide insights to educators and policymakers concerned about teachers' education and professional development; teachers must be equipped with the required technological literacy skills to enhance students' writing in the digital age.

## 1 Introduction

Writing has always been perceived as a daunting and challenging activity for teachers and learners. Writing is a multifaceted task that necessitates individuals to have advanced linguistics and communication skills [1]. However, these skills are not naturally inherited; they are an overwhelming experience that demands deliberate practice in a disciplined setting [2,3] This process becomes more arduous in the EFL context. As a result, a wide range of writing approaches have been developed and embraced by scholars and academics. Specifically, four significant pedagogical approaches were adopted in EFL classes: product, process, genre and process-genre [[4], [5], [6]]. Each of these approaches introduces writing from a different theoretical perspective. Writing approaches provide EFL teachers with guidelines and frameworks to facilitate students learning and promote their writing proficiency.

Despite this, teaching writing remains a challenging process that has proven to be quite intricate for teachers [[4], [5], [6]]. As part of the writing instruction, teachers are responsible for providing students with guidance and support to ensure students can complete writing tasks competently. In this sense, many scholars advocate the significance of academic literacy skills of writing teachers as a crucial element for effective instruction, especially in language classes [7,8].

Within this context, academic literacy works as a multi-dimensional concept that covers the ability to read, write, produce, interact with, and disseminate knowledge in an academic context [9]. Consequently, the development of academic literacy skills appears more demanding today, especially when considering the evolving digital world. Teachers are required to develop literacy skills and pedagogical expertise that are compatible with the ubiquity and diversity of technology advancement [7], a logical reason for teachers to possess multidimensional knowledge.

### 1.1 Teachers' knowledge

Scholars have substantially researched teachers' knowledge. Recent studies have shown that teachers' knowledge of empirically supported writing pedagogies affects students' mastery of writing skills [[10], [11], [12]]. Shulman [13,14] conceptualized the complex nature of teachers' knowledge and depicted how subject matter knowledge and pedagogical knowledge are interrelated. As per Shulman [13,14], for a teacher to be successful, it is vital to possess knowledge in seven domains: knowledge of content, general pedagogical knowledge, curriculum knowledge, pedagogical content knowledge, knowledge of students' characteristics and needs, knowledge of the educational goals and contexts. Nevertheless, pedagogical content knowledge (PCK) stands out as an exceptionally significant domain as it represents the foundational framework of the knowledge that must be gained to comprehend, interpret and personalize the content of the subject in order to accommodate a wide variety of learners' needs [13].

PCK theory encouraged a change in the research agenda to address emergent knowledge that stems from the intersection of content and pedagogy. This viewpoint challenges conventional approaches, treating content and pedagogical knowledge as two distinct constructs [15]. Shulman [14] states that a teacher's competency is determined based on their ability to deal with the subject content and select the most appropriate strategies to make the content relevant and accessible for learners. In writing classes, teachers' efficacy is determined by their ability to diversify writing instructions by introducing multiple text genres and employing the latest strategies to meet learners' needs [16].

With this concern in mind, infusing technology into classroom instruction has become urgently enforced by mounting evidence from the extant literature that proves its positive influence on the quality and quantity of students' writing [[17], [18], [19]]. Qoura [20] argued for the use of multimodal literacies and technology to make writing more complex, appealing, creative and collaborative. According to Boudjadar [21] and Qoura [20], technology motivates learners to communicate their thoughts and share their ideas with a wider audience through [20,21] blogs, social networks, forums and emails. Unsurprisingly, Wang [22] reported that around 70% of the learners believe technology has spurred their motivation to write in English. More research claims that incorporating technology improves students' writing, engagement and motivation [17,23,24] and provides opportunities to receive feedback from a broad range of people, not just their teachers and classmates [21].

Despite this, studies have shown that technology integration in writing classes is neglected [25,26]. According to Applebee and Langer [25], writing classes tend to be teacher-centered when it comes to technology implementation. Using technology in the classroom has raised concerns about how language teachers integrate linguistic content [27].

### 1.2 Technological pedagogical and content knowledge (TPACK) framework

On account of technology advancement, various frameworks and models have been empirically developed to support e-inclusion in classroom instruction [28,29] and support scholars in defining the knowledge and skills needed to infuse technology into classroom instruction [30]. One of the more current frameworks is referred to as TPACK (Technological, Pedagogical and Content Knowledge), which was developed by Mishra and Koehler [15] based on Shulman's [14] PCK theory. TPACK is a model that highlights the link between three core knowledge: content knowledge, pedagogical knowledge and technological knowledge. Furthermore, the modal emphasizes the knowledge that results from the intersection of the three core areas of knowledge: Pedagogical Content Knowledge (PCK), Technological Content Knowledge (TCK), Technological Pedagogical Knowledge (TPK), and Technological Pedagogical and Content Knowledge (TPACK) [31]. According to Mishra [15] and Mishra and Koehler [15], TPACK is the key to effective technology integration as it incorporates the characteristics of the three knowledge domains.

TPACK has been established as a leading framework for defining teacher knowledge in the digital age [[32], [33], [34], [35], [36], [37]]. Consequently, research on TPACK has flourished significantly. Studies suggest that effective teaching is correlated with teachers' capability to incorporate technologies to fulfill the varied needs of their students [27,[38], [39], [40]]. In the field of English language Education, TPACK research has been conducted to measure teachers' TPACK level, TPACK relationship with other factors, TPACK development, TPACK implementation, and TPACK instrument development [27,32].

### 1.3 The Qatari context

Education in Qatar has gone through dramatic fluctuations during the past few decades. Qatar's educational system was criticized until the late 1990s as being antiquated, rigid, and change-resistant [41]. In 2001, an educational reform was initiated through a joint effort between the government of Qatar and RAND, a non-profit research organization [41,42]. The call for reform in Qatar's education system was sparked by the low performance of students on global standardized exams, the prevalence of conventional teacher-centered teaching approaches, and policymakers' desire to enhance the overall quality of education in Qatar across all grade levels from Kindergarten to 12th grade (K-12) [43].

Upon completing the evaluation, RAND noted several deficiencies related to the quality of the curriculum, type of instruction, availability of technology and leadership policies [42,44,45]. In 2002, the initiative “*Education for a New Era”* (EFNE) was launched in response. Following the recommendations of RAND, a standards-based system was created to provide guidance for the development of curriculum, teaching methods and teachers' professional growth [41]. The new system focused on four main disciplines: Science, Math, Arabic, and English. Furthermore, the national professional standards for teachers and school leaders were developed in 2007 to enhance teaching and leadership skills [41].

Along with the shift towards decentralization, additional challenges arose regarding educators' capacity building, the sustainability of reform principles, gaining the acceptance and support of stakeholders, and finding eligible operators [41]. Over time, these challenges and others eventually led to a gradual reversal of the Western-inspired reform initiative, resulting in a shift back to a centralized system officially proclaimed through the Emiri Decree No. 9 in 2016.

### 1.4 Research problem

Despite the mandatory inclusion of English subject from K-12 in Qatar, current instructional practices employed in schools fail to provide the intended outcomes. Qatar's performance has been below average in recent years, based on data gathered from worldwide standardized assessments like PISA and PIRLS [43,[46], [47], [48], [49]]. Moreover, Qatar is considered a nation with a “low proficiency” level in the English language according to English First English Proficiency Index [50].

Additionally, studies indicate that despite the considerable investment made by the Qatari government to boost the sector of educational technology, the desired outcomes of improving classroom access to technology have not been achieved [51]. Furthermore, many educators lack the essential digital competencies required to maximize their teaching methods, advance students' learning, and significantly contribute to the world's evolving digital market [52].

In light of this, the quality of classroom instruction, including writing, has gained scholars' attention. Writing instruction in EFL classes is often teacher-centered, and the use of technology falls below expectations. Teachers frequently use laptops, computers, projectors, smart boards, and e-textbooks to perform various instructional activities such as lesson planning and sourcing visual aids, while learners seldom experience a learning environment that actively integrates technology [53].

According to Blessinger et al. [53], Effective technology infusion in the classrooms requires teachers to possess three domains of knowledge: technological knowledge (TK), pedagogical knowledge (PK) and content knowledge (CK). These domains form the basis of the TPACK model, which guides the successful integration of technology into classrooms. Based on this knowledge, teachers need to make numerous decisions to strike a balance between covering the curriculum adequately and addressing the individual needs of their students [54].

In the last decade, little research has been conducted on TPACK in language teacher education [27]. Hence, this study aimed to explore teachers' TPACK in Qatari preparatory schools, focusing on two questions:

1. What level of TPACK do EFL teachers have in terms of integrating technology into teaching writing to EFL students in preparatory schools in Qatar?
2. Are there any statistically significant differences in EFL teachers' TPACK due to gender, years of experience and the received professional development?

### 1.5 Significance of the study

A substantial body of literature has explored teachers' TPACK, particularly in the disciplines of mathematics, science, and social science. However, there is a scarcity of literature that examines English language teachers’ TPACK [27,38,55]. The studies become noticeably fewer when it comes to EFL-TPACK [39,40,56]. In addition, most TPACK research has focused on pre-service teachers rather than in-service teachers [57,58].

This research contributes significantly to the field of EFL writing due to the paucity of studies that examined teachers' TPACK in writing classes [38,59,60] and specifically in the ESL/EFL context [9,62,63]. Furthermore, to the best of the researchers' knowledge, no other study has been found investigating EFL teachers' TPACK in teaching writing in Qatar.

In addition, assessing EFL teachers' TPACK knowledge could yield valuable information regarding teachers' readiness to integrate technology into their writing instruction in Qatari schools. In turn, this study will help stakeholders design and provide professional development programs based on teachers' needs.

## 2 Methodology

The current study embraced the descriptive research design and collected quantitative data using a self-reported survey. The purpose of the descriptive design is to provide the most accurate description of an actual phenomenon and its characteristics [61]. The survey, used in this study, was adapted from Schmidt's [62] study, which was developed based on Baser et al.’s [63] empirically validated EFL TPACK survey, which in turn was derived from Schmidt et al.’s [64] survey.

### 2.1 Participants

Participants in the study were EFL teachers employed in Qatari government preparatory schools during the academic year 2021. In total, 182 teachers out of 365 responded to the survey, representing a 49.9% response rate. Cohen et al. [65] state that this percentage enables the generalization of the findings and aids in minimizing sampling bias [65]. It is essential to underline that approximately 72% of teachers in Qatari government schools are not Qataris [66]. The workload for EFL teachers is normally from 10 to 18 classes each week, except for English coordinators, who are responsible for five classes in addition to professional and administrative duties. Table 1 below summarizes the demographic profile of the participants.

**Table 1:** Descriptive statistics of demographic characteristics.

Characteristics	Level	Frequencies	Percent
Gender	Male	72	39.6%
	Female	110	60.4%
Highest Degree	Bachelor	130	71.4%
	Postgraduate Diploma	23	12.6%
	Master	25	13.7%
	Ph.D./Ed.D.	4	2.2%
Country of Education	Qatar	25	13.7%
	Others	157	86.3%
Years of Teaching Experience	1–5	12	6.6%
	6–10	43	23.6%
	11–15	47	25.8%
	More than 16	80	44%
Previous Professional Development Related to Integrating Technology into Writing Instruction	yes	109	59.9%
	No	73	40.1%

### 2.2 Instruments

This study employed a two-section web-based survey for data collection. Surveys are a predominant, effective, and convenient tool used to collect descriptive information on a one-time basis regarding the phenomena under investigation [65]. The first section contained demographic data. The second section, comprising 24 items, was developed to evaluate the technological, pedagogical and content knowledge of EFL teachers in writing classrooms. Teachers were required to respond to each item using a 5-point Likert scale. (1 = strongly disagree to 5 = strongly agree).

To better suit the Qatari context and the study's objectives, slight modifications were made to the survey. For example, the acronym *D2L* (*Desire 2 Learn)*, which is a learning platform used in the USA, was changed into *LMS* (*Learning Management System*), a platform used in Qatari public schools. Additionally, the term *foreign language* was used instead of a *second language*. Furthermore, recognizing that CK, TK, and PK are the fundamental areas of knowledge that constitute and influence teachers' TPACK, the survey was designed to focus on these areas [15]. This decision was supported by scholars who argued that it is difficult to draw clear boundaries between the TPACK domains of TCK, PCK, and TPK, which makes them challenging to assess [60,67].

Expert judgment was essential to ensure the survey had both content and construct validity [65]. Six university professors who are experts in the field were asked to review and comment on the survey. In addition, the researchers conducted a survey pilot test with 18 people who did not end up in the final sample [68]. With only a few minor changes needed, mostly related to language, the feedback received on the survey was extremely positive. The researchers revised and modified the survey based on feedback, comments, and suggestions.

In addition, the reliability analysis was performed using Cronbach's alpha to assess the internal consistency of the multiple Likert-type sections of the survey. The reliability values (Table 2) obtained from the survey range from high to average and are considered satisfactory. Research shows that a value of 0.67 or higher is acceptable [65].

**Table 2:** Cronbach's Alpha reliability values.

Section	No. of items	Cronbach's Alpha value
Technological Knowledge (TK)	9	0.86
Pedagogical Knowledge (PK)	5	0.77
Content Knowledge (CK)	6	0.77
Technological pedagogical Content Knowledge (TPACK)	4	0.75
Total TPACK items	24	0.90

### 2.3 Data collection and analysis

In the study initial phase, the researchers sought Qatar University Institutional Review Board's (QU-IRB 1407-EA/20) approval. Before collecting data, teachers signed the consent forms that covered the study purpose, the withdrawal, voluntary participation, non-traceability, anonymity, confidentiality, and considerations of beneficence and non-maleficence.

To collect the data, the researchers decided to use a web-based survey for being cheaper, faster, more convenient, more accessible and more environmentally friendly than the traditional paper survey [65]. The researchers utilized JotForm software to develop the web-based survey due to its adaptability to different uses structures based on the researchers' needs. In addition, using the JotForm enables the researchers to uphold the confidentiality requested by the QU/IRB. Teachers in public preparatory schools received the survey via WhatsApp applications and emails.

Upon receiving teachers' survey responses, the quantitative data underwent analysis using SPSS version 27. Means and standard deviations were computed for items related to teachers' knowledge, and to explore the relationship between variables, the researchers conducted inferential statistics. Particularly, the independent *t*-test was performed to investigate any statistical differences between the independent variables (gender and the received professional development) and the dependent variables (teacher's TPACK). Finally, one-way analysis of variance (ANOVA) was performed to examine the differences between teachers' TPACK and teachers' experiences.

## 3 Findings

Question one: What level of TPACK do EFL teachers' have in terms of integrating technology into teaching writing to EFL students in preparatory schools in Qatar?

The first question addressed participants’ current knowledge regarding technology integration in writing instruction. To determine the level of knowledge, the researchers divided the means into three categories (Table 3) based on the range between the highest and lowest values (5-1 = 4). Then the range was divided by three resulting in a value of approximately 1.33 (4 ÷ 3 = 1.33).

**Table 3:** Level of knowledge according to the means.

Weighted Average	Result Interpretation
1–2.32	Low
2.33–3.65	Moderate
3.66–5	High

Overall, the comparison of TPACK subdomains (Table 4) indicates that teachers possess a high level of knowledge in all four domains, with content knowledge (CK) yielding the highest mean value (*M = 4.29, SD = 0.68*). In contrast, teachers' technological, pedagogical, and content knowledge (TPACK) exhibited the lowest mean value (*M = 3.92, SD = 0.76*), while technological knowledge (TK) and pedagogical knowledge (PK) showed the same mean value *(M = 4.15)*, although they had different standard deviations.

**Table 4:** TPACK subdomains comparison.

Item	Mean	Degree	SD	Rank
CK	4.29	High	0.68	1
TK	4.15	High	0.7	2
PK	4.15	High	0.73	3
TPACK	3.92	High	0.76	4

### 3.1 Technological knowledge

Table 5 presents findings of the first section of the TPACK survey which includes nine items on technological knowledge of teachers. The results reveal that teachers in general possess high level of technological knowledge (*M = 4.15, SD = 0.7*). The item that describes the teacher's professional ability to use content development tools, like office programs and Learning Management Systems (LMS), scored remarkably the highest (*M = 4.48, SD = 0.87*). On the other hand, teachers' responses on their ability to troubleshoot common computer problems independently ranked last (*M = 3.82, SD = 0.1*).

**Table 5:** Descriptive statistics of teachers' technological knowledge (TK).

No.	Item	Mean	Degree	SD	Rank
1.	I can use content development tools like office programs (i.e., Word, PowerPoint, etc.) and Learning Management Systems (LMS) with high proficiency.	4.48	High	0.87	1
2.	I can use digital classroom equipment such as projectors and smartboards.	4.41	High	0.81	2
3.	I can use computer input/output devices such as printers, headphones, scanners, etc.	4.4	High	0.92	3
4.	I can use basic technological terms (e.g., operating system, wireless connection, cloud storage, file sharing … etc.) appropriately.	4.32	High	0.99	4
5.	I can adjust computer settings such as installing software and establishing an Internet connection.	4.07	High	1.07	5
6.	I can use different types of software that help me complete my tasks efficiently.	3.99	High	0.89	6
7.	I can create multimedia (e.g., video, web pages, etc.) using text, pictures, sound, video, and animation.	3.95	High	1.08	7
8.	I can use collaboration tools (wiki, Google Drive apps, blogs, social media, etc.) in accordance with my objectives.	3.95	High	0.98	8
9.	I can troubleshoot common computer problems (printer problems, Internet connection problems, etc.) independently.	3.82	High	0.1	9
	Total	4.15	High	0.7	

### 3.2 Content knowledge

Table 6 displays that all teachers exhibited a strong level of content knowledge. Among the five statements, teachers demonstrated the highest level of knowledge in regards to their understanding of English writing conventions, with a mean score of 4.46 and a standard deviation of 0.74. Conversely, teachers displayed the lowest level of understanding in relation to how rhetoric affects communication, with a mean score of 4.06 and a standard deviation of 0.74.

**Table 6:** Descriptive statistics of teachers' content knowledge (CK).

No.	Item	Mean	Degree	SD	Rank
1.	I understand the writing conventions in English.	4.46	High	0.74	1
2.	I can rhetorically analyze texts written in English.	4.35	High	0.79	2
3.	I can express myself in a wide range of writing genres.	4.3	High	0.77	3
4.	I understand how genre functions in texts written in English.	4.3	High	0.68	4
5.	I understand how rhetoric influences communication.	4.06	High	0.86	5
	Total	4.29	High	0.68	

### 3.3 Pedagogical knowledge

Concerning teachers’ pedagogical knowledge, Table 7 highlights that teachers have a high level of PK. Teachers were in high agreement regarding their proficiency in using appropriate teaching methods and techniques for EFL writing as reflected by a mean score of 4.25 and a standard deviation of 0.85. Conversely, teachers demonstrated a lower level of proficiency in supporting students' self-regulated learning through out-of-class work, with a mean score of 4.05 and a standard deviation of 0.88.

**Table 7:** Descriptive statistics of teachers' Pedagogical knowledge (PK).

No.	Item	Mean	Degree	SD	Rank
1.	I can use teaching methods and techniques that are appropriate for foreign language writing students.	4.25	High	0.85	1
2.	I can integrate the experience that I gain from professional development programs into my teaching process.	4.19	High	0.87	2
3.	I align my teaching practices with the writing lesson's outcomes.	4.18	High	0.78	3
4.	I can design learning experiences that are appropriate for foreign language writing students.	4.12	High	0.9	4
5.	I can support students' learning in accordance with their physical, mental, emotional, social, and cultural differences.	4.12	High	0.87	5
6.	I can support students' out-of-class work to facilitate their self-regulated learning.	4.05	High	0.88	6
	Total	4.15	High	0.73	

### 3.4 Technological pedagogical and content knowledge

The TPACK construct included four items. Table 8 illustrates that the overarching teachers' TPACK level was high with a mean of 3.92 and a standard deviation of 0.76. The first and second items which have the same mean score of 4.02 and share the top rank, indicate that teachers feel capable of supporting students in using technology to become independent writers and using collaborative tools to facilitate writing development. However, teachers’ ability to use interactive tools scores the lowest (*M = 3.76, SD = 1.02)* *(see* *).*

**Table 8:** Descriptive statistics of teachers' TPACK.

Item	Mean	Degree	SD	Rank
I can support students as they use technology to become independent writers and users of English.	4.02	High	0.78	1
I can use collaborative tools (e.g., Google Drive apps, LMS, Voice thread, etc.) to support students writing development.	4.02	High	0.93	2
I can support my professional development by using digital tools and resources to continuously improve my ability to teach foreign language writing.	4.01	High	0.83	3
I can use Web 2.0 tools (interactive presentation software, digital story tools, etc.) to develop students' language and writing skills.	3.67	High	1.02	4
Total	3.92	High	0.76	

Question Two: Are there any statistically significant differences in EFL teachers' TPACK knowledge due to gender, years of experience and the received professional development?

### 3.5 Teachers' knowledge by gender

An independent sample *t*-test was performed to identify any significant differences between male and female teachers. As shown in Table 9, the results indicated significant differences in TK *(p = 0.02*), with male teachers reporting a higher level of TK *(M = 4.30, SD = 0.67)* compared to female teachers *(M = 4.06, SD = 0.71).* Similarly, there were significant differences in CK *(p = 0.04),* with male teachers reporting a higher level of CK *(M = 4.42, SD = 0.70)* than female teachers *(M = 4.21, SD = 0.66).* However, no significant differences were found in PK or TPACK based on gender.

**Table 9:** *t*-test Statistic of Teachers' TPACK According to Gender.

Domain	Gender	N	M	SD	t	Sig.
TK	Male	72	4.30	0.67	2.29	0.023
	Female	110	4.06	0.71		
CK	Male	72	4.42	0.70	2.07	0.04
	Female	110	4.21	0.66		
PK	Male	72	4.22	0.77	1.02	0.31
	Female	110	4.11	0.71		
TPACK	Male	72	4.03	0.84	1.51	0.13
	Female	110	3.86	0.70		

### 3.6 Teachers' knowledge by teaching experience

A one-way analysis of ANOVA was performed to investigate whether the teaching experience had any significant impact on teachers' knowledge. As Table 10 shows, the analysis revealed statistically significant differences between teaching experience and technological knowledge (TK) *(p = 0.05).* A Tukey post hoc test was conducted, which indicated that teachers with 1–5 years of experience scored higher in TK than those with 6–10 or 16 or more years of experience. No significant differences were found between teaching experience and CK, PK, or TPACK.

**Table 10:** ANOVA for teachers' knowledge and practices by teaching experience.

Domain		Sum of Squares	df	Mean Square	F	Sig.
TK	Between Groups	3.845	3	1.282	2.686	**.048**
	Within Groups	84.958	178	.477		
	Total	88.803	181			
CK	Between Groups	1.262	3	.421	.909	**.438**
	Within Groups	82.393	178	.463		
	Total	83.655	181			
PK	Between Groups	1.068	3	.356	.657	**.580**
	Within Groups	96.471	178	.542		
	Total	97.539	181			
TPACK	Between Groups	.146	3	.049	.083	**.969**
	Within Groups	104.773	178	.589		
	Total	104.919	181			

### 3.7 Teachers' knowledge according to professional development

To investigate whether professional development had an effect on teachers' knowledge, an independent sample *t*-test was conducted. The results, displayed in Table 11, indicate a statistically significant difference *(p = 0.05)* in technological pedagogical content knowledge (TPACK) between teachers who received professional development and those who did not. Specifically, teachers who received professional development in using technology to teach writing reported a higher level of TPACK (M = 4.02, SD = 0.80) than their colleagues who did not receive such training (M = 3.79, SD = 0.68).

**Table 11:** Teachers' knowledge and practices according to the professional development.

Domain	PD	N	M	SD	t	Sig.
TK	Yes	109	4.20	0.74	.99	.33
	No	73	4.09	0.64		
CK	Yes	109	4.31	0.75	.33	.74
	No	73	4.27	0.55		
PK	Yes	109	4.17	0.81	.35	.73
	No	73	4.13	0.60		
TPACK	Yes	109	4.02	0.80	2.03	.04
	No	73	3.79	0.68		

## 4 Discussion

The emerging technologies and the changes that the world is going through while trying to cope with the digital transcends put teachers at stake; there is no escape but to develop and sharpen their digital skills to keep the field of education up-to-date, acknowledging the fast-growing and ever-changing world. In this sense, technology is no longer a luxury but a providential tool that should be interwoven in classroom instruction. Therefore, this study investigated the knowledge of EFL teachers in writing classes in Qatari preparatory schools.

The first question examined EFL teachers' perception of their TPACK in writing classes. To be more specific, the evaluation of teachers' knowledge focused on the following four dimensions: TK, PK, CK, and TPACK. Results obtained showed that teachers exhibited a high level of proficiency across all TPACK constructs, claiming high confidence in CK followed by PK, TK and TPACK. The results are in agreement with the studies of Alharbi [69], Alqurashi et al. [70] and Kozikoğlu and Babacan [71], who reported a high level of teachers' TPACK.

Further examination of the highest-ranked items within each domain reveals that they pooled under fundamental skills like using office software, interactive whiteboard and printers, understanding writing conventions, and the ability to use effective teaching methods, etc. It is worth noting that with the availability of technology in Qatari schools, teachers' use of technology hovers around preparing and delivering lessons, with fewer opportunities for students to engage actively with technology [72].

Conversely, items related to the use of advanced multimedia and interactive technologies received the lowest rating. In fact, using these technologies requires active-learning students with higher skills in technology. Thus, it is reasonable to believe that EFL writing classrooms are teacher-centered, as teachers appear to be less self-assured in utilizing interactive and collaborative technologies. Similarly, Cheung and Jang's [60] found that writing classes were teacher-driven. The researchers argue that teachers should gain a more profound knowledge of students' needs to integrate technology into writing instruction better. Consequently, these findings cast doubts on the effectiveness of the pre-service and professional development programs offered to the in-service teachers.

In addition, teachers reported a higher CK among the four dimensions of TPACK than the other subdomains. Therefore, it is highly important to note that around (86.3%) of the teachers have obtained their diplomas outside Qatar; hence, this research outcome might be taken with caution when characterizing teachers’ educational system in Qatar. In addition, it is worth mentioning that effective teaching requires more than possessing a sound knowledge of the content. Teachers must also have strong pedagogical and technological knowledge in order to facilitate successful technology integration in their classrooms [60,73]. Alqurashi et al. [70] reported similar results stating that EFL teachers in Saudi Arabia exhibit greater CK levels compared to other domains of TPACK domains. Of course, it is important to highlight that the manpower in the different sectors in the Gulf States is expatriates [66].

The second question analyzed how gender, received professional development and teaching experience influence EFL teachers’ TPACK. Based on the earlier presented results, the study concludes that EFL male teachers have higher TK than females, which coincides with Ergen et al. [74] meta-analysis study on Technological Pedagogical Content Knowledge by Gender. Could culture play a role in this? In general, technology is considered more of a male interest in the Arab world. Conversely, women generally develop unfavorable attitudes toward digital tools and information technology [75]. These findings converge with other research proving the existence of the global digital-gender gap. Evidence confirmed the superiority of males in developing their technological competencies, whereas females have a low self-perception of technology use [76,77]. Hence, enhancing female teachers' digital literacy to adapt to rapid technological advancement is highly critical.

However, various gender-related studies have yielded different findings [74,78,79], where other reasons attributed to differences in research sample size and characteristics or gender distribution were reported.

Moreover, the results of this study highlighted the impact of years of experience on teachers' knowledge and practice. For example, teachers with 1–5 years of experience demonstrated higher TK than teachers with more years of teaching. One way to reason that could be on a demographic basis where the participants' characteristics could influence the participants' choices. This study reported that about half of the participants have 16 and more years of teaching experience, and around 26% have 11–15 years. It is possible that those experienced teachers are less likely to adapt to the new technologies than younger teachers, who are referred to as digital natives [80]. Drajati et al. [35] reported similar findings indicating that novice teachers were more adept at implementing various forms of technology in their classrooms than experienced teachers. In fact, professional development programs that equip teachers with the necessary digital knowledge and competencies to effectively incorporate technology in writing instruction become more vital in such contexts. This is especially true given the results that reveal a significant difference in teachers' TPACK, with those who received training in technology integration exhibiting a distinct advantage. Similar results are found in Kozikoğlu and Babacan [71]. A large number of experienced teachers completed their education programs prior to many of the current interactive technologies appearing. Definitely, this puts the concept of teachers' sustainability more on call; the overwhelming diversity of emerging technologies requires teachers to uplift their technological skills through continuous sustainability [81]. Effective professional development is a personalized and dynamic process that cannot be approached with a “one-size-fits-all” mentality. It requires ongoing and interactive engagement, allowing teachers to observe new techniques in action and actively participate in their own learning [81]. Teachers are invited to establish their own professional development communities to exchange expertise and benefit from those who know more.

### 4.1 Limitations and future research

Despite the findings of this study, the authors report the following limitations. One considerable limitation is related to the study instrument. Data collected from self-reported surveys could be biased and may not accurately reflect teachers’ TPACK actual practices in writing classes. However, we took steps to ensure the reliability and validity of our survey instrument and the data it produced. We also analyzed the data using appropriate statistical methods to ensure our findings are robust and reliable. Future studies can collect qualitative data through classroom observations and interviews to get a broader perspective on teachers' classroom practices.

The second limitation relates to the scope of the study. The study focused on measuring the core knowledge (TK, Pk, CK, TPACK) teachers should obtain to successfully integrate technology in writing classes. Although the argument for this selection was presented in the methodology section, researchers may study other knowledge types resulting from the intersection of the primary knowledge (TCK, TPK, PCK) to understand better teachers' knowledge in writing classes.

Finally, the participation in this study was limited to in-service teachers in preparatory schools in Qatar. Therefore, TPACK findings cannot be generalized to other populations and contexts. Therefore, future researchers could explore teachers’ TPACK-Writing perspectives in different grades to gain a comprehensive view of teachers' practices in schools.

## 5 Conclusion

In recent years, the TPACK framework has been broadly researched and applied in various contexts in an attempt to understand the facets of teachers' knowledge required for effective technology integration. This study was an effort to explore the TPACK of EFL teachers in writing classes in Qatar. The findings demonstrate that the level of confidence among EFL teachers in Qatari preparatory schools is relatively high in all TPACK dimensions. According to the teachers, CK was higher among the four components of TPACK. However, results show that teachers' use of advanced technology to meet the native digital needs in teaching writing is to be enhanced. Furthermore, the study reveals that gender, years of experience and professional development impact teachers’ TPACK. In particular, male teachers were found to possess a higher level of TK than their female counterparts, and teachers with 1–5 years of experience were the highest in TK. Moreover, teachers who participated in professional development outscored their peers in TPACK.

Based on the presented findings, this study concludes with a few implications. First, the study suggests that policymakers should review learning standards and competencies to ensure that digital literacy is recognized as a fundamental skill. Additionally, the findings may provide guidance for educators in Qatar, particularly those responsible for teacher education and professional development, to prioritize equipping teachers with the digital literacy skills necessary to improve student writing in the age of technology. In addition, it would help to conduct further research on the TPACK of teachers who obtained their higher education programs degrees in Qatar to better understand the quality of the local teacher education programs.

## Production notes

### Author contribution statement

Hanadi Ahmad Abubakir: Conceived and designed the experiments; Performed the experiments; Analyzed and interpreted the data; Contributed reagents, materials, analysis tools or data; Wrote the paper.

Yousef Alshaboul: Conceived and designed the experiments; Analyzed and interpreted the data; Contributed reagents, materials, analysis tools or data; Wrote the paper.

### Data availability statement

The data that has been used is confidential.

## Declaration of competing interest

The authors declare that they have no known competing financial interests or personal relationships that could have appeared to influence the work reported in this paper.

## References

[1] Hidayati K.H. (2018). Teaching writing to EFL learners: an investigation of challenges confronted by Indonesian teachers. Langkawi: J. Assoc. Arab. Eng..

[2] Graham S., Rijlaarsdam G. (2016). Writing education around the globe: introduction and call for a new global analysis. Read. Writ..

[3] Kitchakarn O. (2012). Using blogs to improve students' summary writing abilities. Turk. Online J. Dist. Educ..

[4] Akinwamide T.K. (2012). The influence of Process Approach on English as second language Students' performances in essay writing. Engl. Lang. Teach..

[5] Ho N., Thuy H. (2009). Teaching efl writing in Vietnam : problems and solutions - a discussion from the outlook of applied linguistics. VNU J. Sci. Foreig. Lanug..

[6] Mozaheb M.A., Seifoori Z., Beigi A.B. (2013). Effective Iranian EFL writing teachers (A technology-based framework). Proced. Soc. Behav. Sci..

[7] Fu J., Wang Y. (2022).

[8] Muresan L.M., Orna-Montesinos C. (2021).

[9] Li D. (2022). A review of academic literacy research development: from 2002 to 2019. Asian-Pacif. J. Sec. Foreig. Lang. Educ..

[10] Alabere R.A., Shapii A. (2019). The effects of process-genre approach on academic writing. JEE.

[11] Hussin S.N.L., Abdul Aziz A. (2022). Rethinking the teaching approaches of ESL/EFL writing skills. Int. J. Acad. Res. Prog. Educ. Dev..

[12] Yunus M.M., Chien C.H. (2016). The use of mind mapping strategy in Malaysian university English test (MUET) writing. Creativ. Educ..

[13] Shulman L. (1987). Knowledge and teaching:foundations of the new reform. Harv. Educ. Rev..

[14] Shulman L.S. (1986). Those who understand: knowledge growth in teaching. Educ. Res..

[15] Mishra P., Koehler M.J. (2006). Technological pedagogical content knowledge: a framework for teacher knowledge. Teach. Coll. Rec..

[16] Hodges T.S., Wright K.L., McTigue E.M. (2021). The pre-service teacher self-efficacy for writing inventory (ptswi): a tool for measuring beliefs about writing. Assess. Writ..

[17] Azmi N. (2017). The benefits of using ICT in the EFL classroom: from perceived utility to potential challenges. J. Educ. Soci. Res..

[18] Fidaoui D., Bahous R., Bacha N.N. (2010).

[19] Yunus M.M., Nordin N., Salehi H., Embi M.A., Salehi Z. (2013). The use of information and communication technology (ICT) in teaching ESL writing skills. Engl. Lang. Teach..

[20] Qoura A. (2017). EFL teacher competencies in the ICT age. J. Res. Curr. Instr. Educ. Techn..

[21] Boudjadar T. (2015). ICT in the writing classroom: the pros and the cons. Int. J. Appl. Ling. Engl. Lit..

[22] Wang P. (2011). The effect of computer-assisted whole language instruction on Taiwanese university students' English learning. Engl. Lang. Teach..

[23] Allen L.K., Crossley S.A., Snow E.L., McNamara D.S. (2014). L2 writing practice: game enjoyment as a key to engagement. Lang. Learn. Technol..

[24] Lin S.M., Griffith P. (2014). Impacts of online technology use in second language writing: a review of the literature. Read. Improv..

[25] Applebee A., Langer J. (2011). A snapshot of writing instruction in middle schools and high schools. Engl. J..

[26] Graham S., Capizzi A., Harris K.R., Hebert M., Morphy P. (2014). Teaching writing to middle school students: a national survey. Read. Writ..

[27] Tseng J.J., Chai C.S., Tan L., Park M. (2020). A critical review of research on technological pedagogical and content knowledge (TPACK) in language teaching. Comput. Assist. Lang. Learn..

[28] Pellerin M. (2013). E-inclusion in early French immersion classrooms: using digital technologies to support inclusive practices that meet the needs of all learners. Can. J. Educ..

[29] Rahmany R., Sadeghi B., Chegini A.S. (2014). Normalization of CALL and TPACK: discovering teachers' opportunities and challenges. J. Lang. Teach. Res..

[30] Redmond P., Lock J. (2019). Secondary pre-service teachers' perceptions of technological pedagogical content knowledge (TPACK): what do they really think?. Australas. J. Educ. Technol..

[31] Voogt J., Fisser P., Tondeur J., van Braak J. (2016). Handbook of Technological Pedagogical Content Knowledge (TPACK) for Educators.

[32] Lim P.S., Din W.A., Nik Mohamed N.Z., Swanto S. (Nov. 2021). Current trends in TPACK research in English language Education: a systematic review of literature from 2017 to 2021. Int. J. Educ. Psych. Counsel..

[33] Schmid M., Brianza E., Petko D. (2020). Developing a short assessment instrument for Technological Pedagogical Content Knowledge (TPACK.xs) and comparing the factor structure of an integrative and a transformative model. Comput. Educ..

[34] Bostancıoğlu A., Handley Z. (2018). Developing and validating a questionnaire for evaluating the EFL “total PACKage”: technological pedagogical content knowledge (TPACK) for English as a Foreign Language (EFL). Comput. Assist. Lang. Learn..

[35] Drajati N.A., Tan L., Haryati S., Rochsantiningsih D., Zainnuri H. (2018). Investigating English language teachers in developing TPACK and multimodal literacy. Indones. J. Appl. Ling..

[36] Sarıçoban A., Tosuncuoğlu I., Kırmizi Ö. (2019). A technological pedagogical content knowledge (TPACK) assessment of pre-service EFL teachers learning to teach English as a foreign language. J. Lang. Ling. Stud..

[37] Taopan L.L. (2020). Tpack framework: challenges and opportunities in EFL classrooms. Res. Innovat. Lang. Learn..

[38] Chai C.S., Chin C.K., Koh J.H.L., Tan C.L. (2013). Exploring Singaporean Chinese language teachers' technological pedagogical content knowledge and its relationship to the teachers' pedagogical beliefs. Asia-Pacific Edu. Res..

[39] Debbagh M., Jones W.M. (2015). *Society For Information Technology & Teacher Education International Conference*, Association for the Advancement of Computing in Education.

[40] Wu Y.T., Wang A.Y. (2015). Technological, pedagogical, and content knowledge in teaching English as a Foreign Language: representation of primary teachers of English in taiwan. Asia-Pacific Edu. Res..

[41] Brewer D.J., Augustine C.H., Zellman G.L., Ryan G.W., Goldman C.A., Ryan G. (2007).

[42] Nasser R. (2017). Qatar's educational reform past and future: challenges in teacher development. Open Rev. Edu. Res..

[43] Romanowski M.H., Du X. (2020).

[44] Brewer D. (2019).

[45] Zellman G.L. (2009).

[46] Cruz A.P.S. (2019). PISA 2018 Results what students know and can do. J. Chem. Inf. Model..

[47] Mullis I.V.S., Martin M.O., Foy P., Hooper M. (2017).

[48] Mullis I.V.S., Martin M.O., Foy P., Drucker K.T. (2011). Transplantation.

[49] OECD (2014).

[50] First E. (2020).

[51] Karkouti I.M. (2016). Qatar's educational system in the technology-driven Era: long story short. Int. J. High. Educ..

[52] (2015). ictQATAR, ‘Qatar's National ICT Plan: Advancing the Digital Age’.

[53] Blessinger P., Wankel C. (2013).

[54] Griffith R., Massey D., Atkinson T.S. (2013). Examining the forces that guide teaching decisions. Read. Horiz..

[55] Wetzel K., Marshall S. (2011). TPACK goes to sixth grade: lessons from a middle school teacher in a high-technology-access classroom. J. Dig. Learn. Teach. Edu..

[56] Öz H. (2015). Assessing pre-service English as a foreign language teachers' technological pedagogical content knowledge. Int. Educ. Stud..

[57] Kurt G., Mishra P., Kocoglu Z. (2013).

[58] Koçoǧlu Z. (2009). Exploring the technological pedagogical content knowledge of pre-service teachers in language education. Proced. Soc. Behav. Sci..

[59] Ammade S., Mahmud M., Jabu B., Tahmir S. (2020). TPACK model based instruction in teaching writing: an analysis on TPACK literacy. Int. J. Lang. Edu..

[60] Cheung Y.L., Jang H. (2020). ‘Understanding writing teachers’ technological pedagogical content knowledge. Study Five Serv. Teach..

[61] Atmowardoyo H. (2018). Research methods in TEFL studies: descriptive research, case study, error analysis, and R & D. J. Lang. Teach. Res..

[62] Schmidt N. (2020).

[63] Baser D., Kopcha T.J., Ozden M.Y. (May 2016). Developing a technological pedagogical content knowledge (TPACK) assessment for pre-service teachers learning to teach English as a foreign language. Comput. Assist. Lang. Learn..

[64] Schmidt D.A., Baran E., Thompson A.D., Mishra P., Koehler M.J., Shin T.S. (2009). Technological pedagogical content knowledge (Track): the development and validation of an assessment instrument for pre-service teachers. J. Res. Technol. Educ..

[65] Cohen L., Lawrence M., Morrison K. (2018). http://www.tandfonline.com/doi/abs/10.1111/j.1467-8527.2007.00388_4.x.

[66] Romanowski M.H., Abu-Shawish R.K., Merouani N. (2019). Principals' perspectives on faculty diversity in Qatar's government schools. Educ. Manag. Adm. Leader.

[67] Brantley-Dias L., Ertmer P.A. (2013). Goldilocks and TPACK: is the construct “just right?”. J. Res. Technol. Educ..

[68] Johnson R.B., Christenen L. (2014).

[69] Alharbi M.A. (2020). Exploring the potential of Google Doc in facilitating innovative teaching and learning practices in an EFL writing course. Innovat. Lang. Learn. Teach..

[70] Alqurashi E., Gokbel E.N., Carbonara D. (2017). Teachers' knowledge in content, pedagogy and technology integration: a comparative analysis between teachers in Saudi Arabia and United States. Br. J. Educ. Technol..

[71] Kozikoğlu İ., Babacan N. (2019). The investigation of the relationship between Turkish EFL teachers' technological pedagogical content knowledge skills and attitudes towards technology. J. Lang. Ling. Stud..

[72] Chaaban Y., Ellili-Cherif M. (2017). Technology integration in EFL classrooms: a study of Qatari independent schools. Educ. Inf. Technol..

[73] Mishra P., Koehler M.J. (2008).

[74] Ergen B., Yelken T.Y., Kanadli S. (2019). A meta-analysis of research on technological pedagogical content knowledge by gender. Contemp Educ. Techn>.

[75] Sáinz M., López-Sáez M. (2010). Gender differences in computer attitudes and the choice of technology-related occupations in a sample of secondary students in Spain. Comput. Educ..

[76] Gómez-Trigueros I.M., De Aldecoa C.Y. (2021). The digital gender gap in teacher education: the TPACK framework for the 21st century. Eur. J. Investig. Heal. Psych. Educ..

[77] Gómez-Trigueros I.M., Ruiz-Bañuls M., Ortega-Sánchez D. (2019). Digital literacy of teachers in training: moving from icts (information and communication technologies) to lkts (learning and knowledge technologies). Educ. Sci..

[78] Kazu I.Y., Erten P. (2014). Teachers' technological pedagogical content knowledge self-efficacies. J. Educ. Train Stud..

[79] Wright B., Akgunduz D. (2018). The relationship between technological pedagogical content knowledge (Tpack) self-efficacy belief levels and the usage of web 2.0 applications of pre-service science teachers. World J. Educ. Techn.: Curr. Iss..

[80] Kivunja C. (2014). Theoretical perspectives of how digital natives Learn. Int. J. High. Educ..

[81] Zoch M., Myers J., Belcher J. (2015). Teacher learning in a digital writing camp. J. Technol. Teach Educ..

